# Providing Buffers, Solving Barriers: Value-Driven Policies and Actions that Protect Clients Today and Increase the Chances of Thriving Tomorrow

**DOI:** 10.1007/s40617-023-00876-z

**Published:** 2023-11-20

**Authors:** Teresa Camille Kolu

**Affiliations:** Cusp Emergence, P. O. Box 1796, Berthoud, CO 80513 USA

**Keywords:** Preventive behavior analysis, Adverse childhood experiences, Buffers

## Abstract

Between 1990 and 2018, regions spent between 2.67% (Europe) and 3.6% (North America) of their GDP to treat harmful behavioral, medical, and other effects of significant adverse experience (Bellis et al., 2019 *The Lancet Public Health*, 4(10), e517–e528). Although dose-dependent exposure to adverse childhood experiences harms long-term medical health (e.g., Anda et al., 2006; *European Archives of Psychiatry & Clinical Neuroscience*, 256, 174–186, Anda et al., 2008; *American Journal of Preventive Medicine*, 34(5), 396–403,  Dong et al., 2004; *Circulation*, 110(13), 1761–1766, Felitti and Anda, 2009), six specific buffers (nurturing relationships; nutrition; physical activity; sleep; mental health support; and reducing stress) protect against these harmful health impacts (Purewal et al., 2016, *Zero to Three*, 37(1), 10–17). However, barriers related to access, information, resources, or behavioral needs prevent many from experiencing the benefits. This article describes an approach in which each buffer area is addressed in the context of its overlap with behavior analytic practice, and supported by related policy suggestions. Providers are invited to adopt an informative buffer policy as an antecedent to client services; establish a collaborative network of providers and resources; and expand buffer promotion beyond clients to other stakeholders including caregivers and staff. The aim of this article is to inspire and empower individuals to use several specific actions: (1) learn about buffers and consider barriers to them; (2) educate others about buffers and barriers to them; (3) scan a client’s environment for buffers and barriers; and (4) consider ways to install buffers and resolve barriers for clients or others as appropriate.

## Introduction

Significant aversive stressful experiences have deleterious health impacts (Brady, 1958), which are now broadly recognized (Anda et al., 2006; Anda et al., 2008). Large-scale studies have documented how a host of varied and debilitating medical conditions (see Table 1 for research related to some examples) are compounded and accelerated by dose-dependent exposure to adverse childhood experiences (e.g., Anda et al., 2006; Anda et al., 2008; Dong et al., 2004; Felitti & Anda, 2009). Buffers, on the other hand, are six specific areas (e.g., the nurturing relationship, nutrition, physical activity, sleep, mental health, and reducing stress; see Harris, 2018; and Purewal et al., 2016) in which action is documented to exert effects that mitigate the risk of, and in some cases prevent, the biological damage caused by cumulative exposure to prolonged, inescapable, and distressing aversive and adverse experiences (e.g., Maier & Seligman, 2016; McEwen, 2000, 2004, 2005; Slopen et al., 2014). Behavior analysts and advocates are uniquely positioned to intervene during critical and early points in the lives of countless families already affected by significant stress and adversity, and ethically tasked with minimizing harm for clients and mitigating barriers to effective treatment (Behavior Analyst Certification Board [BACB], 2020). Thus, this article aims to inspire and empower individuals to use several specific actions: (1) learn about buffers and consider barriers to them; (2) educate others about buffers and barriers to them; (3) scan a client’s environment for buffers and barriers; and (4) consider ways to install buffers and resolve barriers for clients or others as appropriate.

**Table 1:** Problems with health outcomes linked to previous adverse experiences

Risk or problem faced after trauma	Reference(s)
Increased likelihood of disease in adulthood (including obstructive pulmonary disease; ischemic heart disease; autoimmune disease; and much more)	Koita et al. 2018; Anda et al., 2008; Dong et al., 2004; Dube et al., 2009
Mental health needs; greater risk of depression	Bethell et al., 2014; Chapman et al., 2004; Huntington & Bender, 1993; Maag & Reid, 2006
Greater number of infections	Wyman et al., 2007; Lanier et al. 2010
Developmental and learning delays and difficulties	Enlow et al., 2012; Strathearn et al., 2001; Burke et al., 2011
Dental problems	Bright et al., 2015
Asthma	Wing et al., 2015; Kozyrskyj et al., 2008; Lange et al., 2011
ADHD/Conduct disorder	Morgan et al., 2016
Sleep disturbances and difficulties	Armitage et al., 2009; Hairston et al., 2011; Wolke & Lereya, 2014
Obesity	Suglia et al., 2012
Suicide related behaviors	Rhodes et al., 2012
Pain (and altered pain perception), gynecological disorders	Paras et al., 2009; Reissing et al., 2003
Toileting difficulties	Nijman et al., 2005
Neurobiological changes that alter attention, handling stress, and more	Karmel & Gardner (1996); Danese & McEwen (2012); De Bellis et al., 1999

In general, buffers are factors that have a protective effect when someone is facing adverse experiences, or recovering from those already faced. Purewal et al. (2016) described an integrated pediatric care model in which screening for ACEs (adverse childhood experiences) leads to specific and coordinated actions by collaborative health-care professionals. They recommended emphasizing six things found in the literature to lower the risk of long-term health concerns (Khoury et al., 2015; Lopresti & Drummond, 2013; Miller et al., 2014; Simkin & Black, 2014; Slopen et al., 2014). These six buffers are: (1) a nurturing relationship; (2) nutrition; (3) regular physical exercise and activity; (4) healthy sleep; (5) mental health support; and (6) mindfulness-based or other stress-relieving practices (e.g., Purewal et al., 2016). Research shows that although children who faced early adverse life experiences face greater risk for diseases, medical problems and challenges, buffers diminish that effect and contribute to the reasons some children succeed in the face of difficult backgrounds (Dube et al., 2009; Felitti, 2002). Buffers can be added to someone’s repertoire or environment at any time using procedures that are conceptually systematic with respect to behavior analysis and already well-integrated in the repertoires of behavior analysts and educators. Unfortunately, for many individuals and families, significant and varied barriers block access and decrease utilization of potentially life-changing interventions.

In addition to poor health outcomes linked in a dose-dependent way to exposure to ACEs, there is significantly compromised brain development for children faced with early adverse experience (Behen et al., 2009; Eluvathingal et al., 2006; Hanson et al., 2013). However, recent research suggests the neural changes accompanying some adverse childhood experience may be partially reversible. In randomized clinical research on the brains of children living in or removed from conditions of neglect, those children placed into a high-quality and stable family environment showed change in several brain regions as a function of stable foster care placement, as detected by magnetic resonance imaging scans (Bick et al., 2015). Bick et al. (2015) randomized infants from Romanian orphanages into a clinical trial of foster care to examine differences between brains that experienced stable foster care placements or childhood in the orphanage. Results indicated that removal from conditions of neglect into a stable family environment changed (and even normalized) brain growth trajectories for the children followed by the study. Inclusion of control children never in orphanages or foster care revealed that the brains of controls were similar in development to those of children in the stable, long-term foster care with trained caregivers. Although the only buffer directly manipulated here was the addition of a stable relationship with caregivers trained to meet the special needs of children from a deprived background, this study implies that some environmental interventions can change the trajectory of neural development even after significant early adverse conditioning and childhood experiences. Buffers, then, are things that can be added to someone’s routine which could potentially mitigate harm from experiences that already took place—in addition to protecting against the effects of future adverse experiences that one might face.

Because several concepts may seem similar and related to the term *buffers*, readers are invited to consider why this term is selected when others might suffice. As summarized by Harris (2018), action within six specific buffering areas engages several biological mechanisms (reducing stress hormones, reducing inflammation, enhancing neuroplasticity, and delaying cellular aging) that affect health and mediate protection from disease onset (Harris, 2018). This specificity (both in terms of the six buffer areas and their effects described by the medical community) distinguishes buffers from other more nebulous concepts such as *protective factors*, *resilience*, and *promotional factors* used in literature on preventing and/or managing the harmful effects of trauma. In contrast to *buffers*, other terms (discussed briefly below) are used in various ways, do not accompany a specific and consistent set of practices or recommendations for clients, and may be accompanied by interpretation problems. For these reasons, perhaps none of these other terms holds as much utilitarian potential as the word *buffers* in conveying the importance of practicing (or solving problems related to) access to six specific areas already important to human beings. First, consider the word *resilience* or the phrase *resilience factors* as a possible alternative to *buffers*. Described in various ways depending on the source and the context, *resilience* involves both the capacity for, and process of, overcoming stress and adversity (Russo et al., 2012; Rutter, 2012). Discussing different definitions, theory, and challenges involved in an interdisciplinary perspective on resilience, Southwick et al. (2014) call resilience alternatively a trait or capacity, a process, or an outcome. The process of resilience may be either constrained by stress (see Rutter, 2006) or spurred by “positive aspects of the environment” (Ungar, 2013), and involves the interaction of developmental, genetic, epigenetic, or environmental factors (Wu et al., 2013). It is important to note that two items mentioned by Wu et al. (2013)—control over stressors (Feder et al., 2011) and connections to supportive adults (see (Burt & Paysnick, 2012)—overlap with the current list of six buffers. In the resilience literature, these two environmental buffers constitute ways the environment can contribute the “active nurturance” so crucial in the development of resilience (see Ungar, 2013).

Buffers also overlap as a concept with *promotive factors* and *protective factors* (see Crouch et al., 2019). Promotive factors are said to include characteristics, assets, or resources that may be situational, social, or individual (Fergus & Zimmerman, 2005; Zimmerman, 2013) and potentially interrupt a problematic life trajectory (Crouch et al., 2019). However, although the two phrases are used interchangeably, resilience theory distinguishes: “promotive factors are called protective factors to distinguish them from promotive factors that only compensate for risk exposure” (Zimmerman 2013). Another difficulty in using “protective factors” concerns the questions, how many protective factors are there, and what are they? This information is important in understanding which practices should be prioritized as most helpful, given the wide range of recommendations provided to consumers of information about resilience and protective factors. For instance, Crouch et al. (2019) studied the relationship between health, ACEs, and whether a person had experienced safe and stable relationships, determining that a safe stable relationship was a “protective factor” that builds resilience and potentially moderates the impact of adverse childhood experiences. However, searches for “protective factors” in major resources to which consumers often turn, result in long lists of “protective factors” that differ depending on the source. The National Center for Injury Prevention & Control (2023) covers the more overtly evidence-based “families who create safe, stable and nurturing relationships” in its list of around 23 factors, but also includes items that may seem inaccessible and nebulous (e.g., “families where caregivers have college degrees or higher”; “children who do well in school”; and “communities where violence is not tolerated or accepted”). A similar source, the Child Welfare Information Gateway, states, that there are six protective factors, listed as nurturing and attachment, knowledge of parenting and of child and youth development, parental resilience, social connections, concrete supports for parents, and social and emotional competence of children. Still others name a different subset of “protective factors”; see Ungar’s article on resilience, which states that there are seven: “access to supportive relationships, opportunities to experience a powerful self-definition, experiences of efficacy, experiences of social justice, access to material resources like food, education and housing, a sense of cohesion within one’s family, community or school, and cultural adherence.” Although it may be helpful to list a small number of factors, these lists are problematic in the sense that they widely differ (with the exception of the near-universal inclusion of safe nurturing relationships, discussed as one critical buffer in this article). It is unclear in the literature whether protective factors are best described as synonymous with resilience or as a factor that contributes to it (see Ungar, 2013).

Overall, the concept of buffers covers six specific things that can be interpretable across cultural lines and that relate to meaningful health outcomes being researched by the medical community, whereas other terms such as protective factors and resilience factors may not be as meaningful. In the current article, buffers are never discussed as *preventing* trauma (as *protective factors* sometimes are), but as potentially preventing and/or mitigating some of the well-documented health harms that result from adverse experiences. Including specificity about the context is critical to a meaningful conversation about harm mitigation. Even as clearer definitions and robust research programs are sorely needed on this concept, a productive starting point for discussing the research and practices presented here is the term *buffers* as it is discussed by Dr. Nadine Burke Harris (2018).

## Buffers as a Preventive Strategy Affecting a Human Rights Issue

The surgeon general’s report on ACEs and resilience (*Roadmap for Resilience*, Bhushan et al., 2020) lists buffering strategies as primary prevention strategies, describing a host of ways to “target healthy individuals and aim to prevent harmful exposures from ever occurring” (p. xxix). Although it is not always considered a responsibility of behavioral providers to enact these buffers, and funding opportunities do not always provide coverage for preventative efforts, this article and a growing body of preventative literature in the medical arena suggests that this intersection constitutes a timely and crucial opportunity for behavior analysts to partner heartily with other practitioners in reducing harm to today’s children, and preventing harm to future generations (described in Purewal et al., 2016). Preventing the well-documented harms of adverse experiences is a human rights issue, with huge inequities in the distribution of information about, as well as access to, protective factors. Around the world, evidence is mounting that policy related to buffers could be of use to countless people. In Australia, around 72% of children have been exposed to one or more ACES (Emerging Minds, n.d.), whereas in India one out of two study participants reported child maltreatment (Fernandes et al., 2021). See Hughes et al. (2021) for prevalence of ACES across 28 European countries. Although numbers range considerably (Hughes et al., 2021), no country escapes this massive burden on children or the high economic costs resulting from it. Worldwide and regional policy initiatives are built on the 1998 ACES studies from the United States (e.g., Felitti, 2002), but resources must be adapted to the kind of challenges disproportionately affecting those with region-specific risk factors (e.g., children whose parents are from a refugee-sending country; persons belonging to a historically marginalized group or First Nations peoples; living in an area affected by natural disasters or in an area that is very remote).

## Sharing and Acting on Knowledge of Buffers Is an Ethical Responsibility

Of primary importance in this discussion of mitigating harm are these two observations: (1) behavior analysts are tasked to prevent harm; and (2) it is difficult to act to prevent something (or to provide its antidote after the fact) if one is unaware of it. At the same time, just as ignorance of the *Professional and Ethical Compliance Code for Behavior Analysts* (BACB, 2020) is not an excuse for failing to practice according to its guidelines, ignorance of how one is causing harm (or failing to do simple actions that mitigate the risk of harm to which someone is already exposed) may not excuse someone from responsibility. It is the responsibility of a behavior analyst to learn about, document, evaluate and minimize the potential risks (see BACB, 2020, p. 5, for a brief summary of ethical decision making to consider risk of harm) related to one’s own actions across the entire scope of services, including accepting clients only within one’s scope of competence; considering medical needs; selecting and using assessment procedures; selecting, utilizing, and evaluating the potential risks and benefits related to, and results of, behavior-change interventions; and providing supervision to others related to the client and their needs (BACB, 2020, 1.05, 2.12, 2.13, 2.14, 2.18, 4.02, 4.04). In particular, the code item on providing effective treatment states that in addition to providing services conceptually consistent with behavior principles, behavior analysts design services that “protect all *clients, stakeholders, supervisees, trainees, and research participants* from harm” (BACB, 2020, 2.01; emphasis in original).

Both protection from harm, and prevention of new harm, are consistent with the *Professional and Ethical Compliance Code for Behavior Analysts* (BACB, 2020). However, for many reasons discussed later as “barriers,” recipients of behavior analysis services may not currently benefit from enriching engagement in the buffering areas, yet each buffer constitutes an area of practice by behavior analysts. Addressing barriers that interfere with engagement in these areas can address conditions that interfere with service delivery (BACB, 2020, 2.19).

## Buffer Areas Described in the Context of Behavior Analytic Literature

The behavior analyst professional is well-positioned to arrange supportive contingencies, environments, and repertoires, and to facilitate fluency and habits with respect to engagement in each buffering area described below. Each buffering area is discussed in turn below, together with brief recommendations for analysts interested in incorporating the area into their practice with clients. Following the six buffers, recommendations and related policy suggestions are provided.

### Buffer 1: The Nurturing Relationship

Having a nurturing relationship and experiencing relational health (Frameworks Institute, 2020) is the gateway through which other needs are met and the primary source of protection or “buffering” against the harmful effects of any adverse experiences (Garner et al., 2021). When adverse experiences take place in the presence of a buffering relationship, the physical and neurological accompaniments to them (e.g., stress) occur and largely subside (unless the experiences are uncontrollable and/or sustained and highly aversive). A “buffering” relationship is important throughout life, but indispensable when the organism is dependent on a caregiver. For many people receiving behavior analysis services, the presence of a caregiver throughout the lifespan remains an important part of the behavioral and environmental arrangements necessary for contacting other reinforcers the client needs, several of which may be related to other buffers including sleep, nutrition, and exercise. Because a caregiver may facilitate the environmental and social support that contributes to other buffers (e.g., healthy sleep habits, nutrition, exercise, receiving mental health care, and using skills to gain calm), it is helpful to educate caregivers on the importance of buffers for themselves and the client, perhaps starting with removing any barriers to the health of this nurturing relationship, and installing needed repertoire components.

The social relationship has been addressed behaviorally since behavior analysis’s early days, when social reinforcement was discussed in terms of generalized conditioned reinforcers used as a means to change behavior (e.g., Skinner, 1953). Later, Iwata (1982/1994) documented the importance of social contingencies in functioning to maintain behavior. Behavior analysts regularly target goals or strategies that address relationships among peers (Fox et al., 2010), family members (Coyne et al., 2020), educators and students (Reinke et al., 2007) or caregivers and their clients (Taylor et al., 2019), solving issues in the relationship to make interaction more reinforcing and successful for either member of the relationship. In behavioral parent training, parents are taught to praise, describe, and imitate appropriate play or talk and be enthusiastic (e.g., Reitman & McMahon, 2013), similar to training for therapists using rapport to increase instructional control. Rapport or presession pairing procedures are related as they are “intended to affect the therapeutic relationship” (Lugo et al., 2017). However, rapport within the therapeutic context is transactional, described as an antecedent-based strategy used to reduce the aversiveness of the therapeutic context (Carbone et al., 2007) and helpful in reducing problematic behavior (Smith, 2001; Sundberg & Partington, 1998; McLaughlin & Carr, 2005).

At the earliest stage of the relationship, a child needs an available caregiver responding to their cues (see Termini & Golden, 2007, for a description of some of the important behaviors that result in sustained and predictable closeness between a parent and child). Later, for the relationship to be safe and healthy, caregiver interactions must also include appropriate content and occur at sufficient rates. Research referenced by proponents of “a buffering relationship” in mitigating adverse experience-related harm (Bethell et al., 2019; Weisleder et al., 2016) suggests that early in the life of both humans and nonhumans, sufficient rates of nurturing and affection behaviors (e.g., the counterparts of licking and grooming behaviors in animals) are crucial for supporting the needs of the developing nervous system, facilitating hormonal regulation instead of the toxic stress experience that occurs outside the presence of this buffer. These nurturing behaviors are sometimes grouped under “positive parenting” skills although the specific parent behaviors vary between literatures; see “positive parenting” as described by Brown et al. (2020) for a perspective outside of behavior analysis in which positive parenting behaviors are characterized and relate to protection against toxic stress versus specific groups of behaviors (see, e.g., Berard & Smith, 2008) in teaching parenting skills to vulnerable families. Positive parenting skills across disciplines involve simple activities such as (for example) talking to children, reading aloud and playing with children (Weisleder et al., 2016) and responding appropriately to child behavior, all teachable skills that behavior analysts have focused on for decades (see Hart et al., 1997; Berard & Smith, 2008).

Behavior analytic work on behaviors related to relationships has sometimes been more transactional than nurturing, guided more by a pathological approach to reducing challenging behavior or as a means to increase language than by a value of nurturing a relationship for its sake. This is not always the case, however, as shown by Shea et al. (2020). In that study, authors addressed responsive caregiving (as it contributes to nurturing care) as a World Health Organization-identified global health priority, in their evaluation of self-guided behavioral skills training delivered in an online asynchronous format. This article was important for several reasons, a few of which are suggested here. First, through publishing their small study, Shea et al. (2020) educated behavior analyst readers about an issue important globally that analysts care about individually (e.g., promoting nurturing care environments). Second, the study modeled how to connect a global priority unrelated to a specific client goal, to the repertoire of behavior analysts and others interested in preventive and supportive topics or interventions enhancing lives in the global community. Third, it used a conceptually systematic intervention, employed in a format that could be disseminated at the population level, because it required no coaching or feedback from an implementer (at least in their small proof of concept study). Other examples of more nurturing (and even joyful) behavior analytic approaches to building responsible and responsive relationships are detailed in works summarizing acceptance and commitment based or constructional approaches to parenting and caregiving (see, e.g., Coyne & Murrell, 2009; Ala’i-Rosales & Heinkel-Wolfe, 2021).

Recommendations for behavior analysts interested in the relationship buffer for their clients relate more to honoring any special relationship that already exists for the client, than doing something new (although resources exist for analysts needing to establish client or caregiver—or their own—skills in this area). All providers working with a client should be informed about the primary nurturing relationships in which the client participates, so that neither therapy, scheduling, nor therapist-driven interactions erode those nurturing relationships. Clarifying roles (and education on the ethical and interpersonal reasons) for providers and the array of team members may also be helpful, because harm and ethical violations can result from unclear communication about a client’s “friends” who are also the client’s advocate, therapist, etc. In some cases, caregivers previously paired with previous aversive control may need special support to reestablish themselves as signals of safety (see Rajaraman et al., 2022) and potentially to abandon practices that broke the trust of their family member in services, or caused physical or other harm. If further resources are needed, the behavior analyst can enlist the support of a socioemotional therapist or related trainings; expand one’s knowledge about child development to support the relationship in appropriate ways; or consider gaining mentorship in working with individuals affected by significant adverse childhood and conditioning experiences.

### Buffer 2: Nutrition and Healthy Eating

Similar to nurturing relationships, nutrition is a global health concern (World Health Organization, 2021). Although feeding and eating disorders affect high numbers of people in the general population (Qian et al., 2022), the numbers are much greater for clients who may be eligible for behavioral services: reported prevalence of feeding difficulties ranges from about 20% to 30% for infants and toddlers (Romano et al., 2015), 40% to 80% for people with developmental disabilities, and nearly 90% for children diagnosed with autism spectrum disorders (Kim et al., 2008). Food refusal or related difficulties can constitute a complex problem with medical and life-threatening complications (Bandini et al., 2010; Gale et al., 2011), requiring the services of those with specialized training and the support of a team of interdisciplinary professionals (Volkert et al., 2016). Behavior analysts have long participated in the assessment and treatment of food refusal (Riordan et al., 1984) and food selectivity (Levin & Carr, 2001; Peterson et al., 2016), as well as assessing caregiver-related variables in children’s behavioral environments (Klesges et al., 1983) or characteristics of the individual; see applications of delay discounting to food-related behavior related to eating behavior and obesity (Weller et al., 2008; Deshpande et al., 2019). In other cases, a constructional approach may be taken early to increase appetitive responses to food and related stimuli through shaping, widening stimulus and response classes, and honoring client assent (see Cihon, 2015). Elsewhere in the literature, community-based programs have focused on increasing consumption (Horne et al., 2004; Horne et al., 2009) or purchasing (Wagner & Winett, 1988) of healthy foods, as well as reducing purchases of high fat foods (Winett et al., 1991). Behavioral interventions have included teaching mindful eating (e.g., Higgs, 2015), using stimulus equivalence to improve the accuracy of estimating portion sizes (Hausman et al., 2014), incorporating rewards and peer modeling (e.g., Horne et al., 2004), environmental arrangement (Horne et al., 2009), modeling and feedback (Winett et al., 1988), self-monitoring and goal-setting (Winett et al., 2007), and using functional analyses-derived procedures to address an array of challenging behaviors that occur related to meals (Stickney & Miltenberger, 1999).

Given the enormous rates of feeding challenges in clients of behavior analysis services, the buffers approach should involve a provider check-in about meals and related behavior preventively, following up by making available an array of resources that improve families’ access to nutrition related supports even when food related challenging behavior is not a concern (or while clients wait for behavioral services). Such access might include connections for each client or family to available resources for expanding their access to nutritious foods regardless of income levels; preventive education on how to tell when a specialist in this area is needed; education for caregivers on how they can prevent food related problems; and information on accessing preventive programs or resources available locally or online for those interested in building a foundation of appetitive skills related to food.

### Buffer 3: Physical Activity

Closely related to nutrition are behaviors involved in regular physical activity, a focus of recommendations by the U. S. Department of Health and Human Services (U. S. Department of Health and Human Services, n.d) given the link between death and a lack of exercise. Health and fitness may be addressed in behavioral ways through task clarification, contingency management, self-monitoring, accountability and feedback, and stimulus control interventions (BACB, 2023). Behavior analytic research has examined the influence of adult interaction and attention (Zerger et al., 2016; Nieto & Wiskow, 2020), peer interaction (Zerger et al., 2017), outdoor activity context (Hustyi et al., 2012), exergaming (Fogel et al., 2010), token reinforcement (Patel et al., 2019), contingency contracting (Stedman-Falls & Dallery, 2020), and self-monitoring, goal-setting, and feedback (Normand, 2008) on physical activity. Given the benefits of physical activity (Lang et al., 2010) and the rates of movement in people with autism (Pan & Frey, 2005), researchers have suggested physical activity be universally prescribed for individuals in this group (MacDonald et al., 2011). Similar prescriptions are recommended for other populations with whom behavior analysts work, given that individuals with developmental disabilities are less physically active than peers (CDC, 2015) and because COVID-19 has led to decreases in levels of physical activity (Oliveira et al., 2022). A recent review of research reinforcement-based behavioral interventions showed that most studies examined failed to produce or measure long-lasting treatment gains (Rotta et al., 2022), suggesting that there is much work to be done in interventions that incorporate physical exercise regularly in the lives of clients who need it. Recommendations for interested behavior analysts include joining a special interest behavior analytic group related to this area; connecting to community programs that can provide a range of low-cost and free options to clients and families before a related behavioral problem appears; and learning about the risk factors affecting their specific population and physical activity. In terms of incorporating physical activity as a lifelong buffer, it may be that beginning earlier with schedules exposing young children and their families to enjoyable and accessible forms of physical activity in their everyday social settings could facilitate a needed shift in this area.

### Buffer 4: Healthy Sleep

Sleep concerns affect high numbers of children, with reported prevalence rates estimated at between 20% and 30% for children who are typically developing (see Johnson & McMahon, 2008) to 50% to 80% for children who are diagnosed with neurodevelopmental disabilities (Kotagal & Broomal, 2012). Across the lifespan, sleep disturbances affect the health of individuals and families (Liu et al., 2018; Luiselli, 2021), the workplace (Crain et al., 2019), and society (Worley, 2018). The consequences of poor sleep range from health risks (Colton & Altevogt, 2006), impaired neurocognitive functioning (O’Brien, 2013) and daytime challenging behavior for the individual (Eshbaugh et al., 2004), to the occurrence of stress (Meltzer & Mindell, 2007) for others affected by the poor sleep of someone in their lives.

In 2021, Luiselli summarized behavior analytic contributions to the assessment and treatment of sleep related behaviors and problems for children and youth, highlighting key issues that are suggested and addressed by researchers in this area. Luiselli’s (2021) review described the conceptual basis for several behavioral interventions and suggested that for maximum effectiveness, sleep interventions be assessment-informed and related to controlling variables. Multifaceted interventions require component analyses, given the interaction between elements including (for example) the classical conditioning involved as one falls asleep (e.g., the physiological state of sleep deprivation; see Piazza & Fisher, 1991) and social variables such as consequences for behaviors that may facilitate or impede sleep and sleep routine-related behaviors (Kuhn et al., 2019). Both antecedent -based and consequent interventions are covered by behavior analytic literature (see Jin et al., 2013; McLay et al., 2019) addressing such sleep-related issues as delayed sleep onset and night waking (Mindell et al., 2015), insufficient hours of continual sleep (van Deurs et al., 2020), sleep disruption (Jin et al., 2013), and excessive daytime sleepiness (Friedman & Luiselli, 2008).

Overall, many antecedent, consequence, and stimulus control related interventions (Luiselli, 2021; Blampied & France, 1993) are available to address an array of problems related to sleep. Recommendations for addressing sleep as it relates to buffering against the harms conferred by adverse experiences, however, should also consider that sleep needs to be both safe and available for both caregivers and clients, and that sleep itself can interact with other behavioral needs and buffers. Carrow et al. (2020) described ways to teach adults to arrange safe sleeping environments for infants, basic skills with which any behavior analyst working with clients of early intervention should be familiar. There are reported cases in the literature in which manipulating duration or other sleep variables affected other behaviors that occurred at different times of the day (see Cautilli & Dziewolska, 2004), or in which health related problems occurred at night and disrupted sleep, school performance and caregiver work (Diette et al., 2000). Thus, a recommendation for applying buffer theory is to use the buffers (and especially sleep) as proxies for health symptoms important to monitor when problematic behavior becomes variable (or to guide further investigation when the activity in the buffer area itself changes, such as when sleep patterns, eating, physical activity, or mental health symptoms shift); see May and Kennedy (2010) on interactions related to problems in health and behavior among people with intellectual disabilities.

Beyond basic skills in sleep health (and safe sleeping environmental arrangements) appropriate to the analyst’s common client population (e.g., Abel et al., 2017, for sleep recommendations and resources when autism is involved), other guidance for behavior analysts interested in sleep as a buffer could include gaining familiarity with factors involved in providing appropriate and individualized recommendations for a client given their age, activity level, and cultural and familial needs; collecting data on how sleep and challenging behaviors interact given a client’s diagnoses, medical and mental health needs, and other areas of buffer health; and establishing connections with a behavior analyst who is skilled in the treatment and assessment of sleep related challenges, for times when a more specialized approach or consultation is needed. Familiarity with local agencies and practices for assessing medical components of sleep challenges is also recommended, so that an analyst can offer timely connections to a client and their family, and collect appropriate baseline data useful to other practitioners and sleep support specialties.

### Buffer 5: Mental Health Care

Having serious mental illness is a risk factor for poor health outcomes, and mental illness reduces life expectancy by an estimated 15 years (Thornicroft, 2013). Issues with mental health overlap strongly with the needs a behavior analyst addresses (Cooper et al., 2015), and affect most populations a behavior analyst serves. For example, although about 1% to 3% of people have an intellectual or developmental disability and about 40% of those experience comorbid mental health disorders, few of those individuals receive specialized mental health services (see Munir, 2016). Participants with mental health related issues played a major part of the historical development of applied behavior analysis (Harvey et al., 2009), but there exist major gaps in the literature (and in the general understanding of the appropriateness of) applying advancements in behavior analysis to the assessment and treatment of challenges in people diagnosed with mental health issues (see Brodhead et al., 2018, for a discussion on aspects of training necessary, and scope of competence problems, for behavior analysts treating the special population affected by mental illness). In fact, applying behavior analysis to mental health issues is not necessarily the goal of the mental health buffer; rather, the behavior analyst is urged to attend to the client’s mental health as an important goal the client may be working on using other resources.

Recommendations for behavior analytic providers who wish to integrate the mental health buffer could include some of the following: distinguish between symptoms of mental illness and behavioral challenges related to other disorders or operant contributions to a client’s behavioral needs; partner with interdisciplinary teams to generate criteria for helping a behavioral services client through crises that necessitate more specialized support, and train behavioral technicians to discriminate between situations involving (for instance) the side effects of behavioral treatment and those requiring additional support; train behavioral providers to recognize and interpret caregiver needs and difficulties as they may relate to mental illness and mental health needs before attempts to modify the caregiver’s behavior through (for instance) parent training, rules, or threats to discontinue therapy or move the service location; help caregivers connect to mental health providers and support networks and resources (making this information available to all clients and caregivers as preventive community networking even if the caregiver does not ask); document what a mental health crisis looks like for a client of behavioral services who has comorbid mental health concerns; and gain the skills needed to recognize possible medical and mental illness-related symptoms of adverse experience, so that a client’s unsafe experiences, new or ongoing experiences of abuse, mistreatment, neglect, or exploitation are not ignored and mistaken either for mental illness or “behavioral needs.”

As the behavioral provider applies these nuances to one’s own practice (individualizing the recommendations for the provider’s population, typical experiences, and cultural context), it is still valuable to learn how a client’s mental health could be supplemented with approaches that are within the scope of competence for a behavior analyst with related expertise (e.g., in acceptance and commitment training, see Biglan et al., 2008; Livheim et al., 2015). This relates to the final buffer, targeting skills that allow experiencing calm and relief from stress.

### Buffer 6: Mindfulness and Reducing Stress

The sixth and final buffer would ensure both client and caregiver have access to skills that effectively result in relieving stress and gaining calm. To this end, several approaches with evidence from both behavior analysis and psychology may be relevant starting points for behavior analysts. The first is using principles related to acceptance and commitment training to establish skills effective in accepting and managing the experience of stressful situations. For example, programs using acceptance and commitment therapy have resulted in lowered levels of stress while increasing mindfulness related skills for adolescents (Livheim et al., 2015) as well as adults (Bethay et al., 2013; Bond & Bunce, 2000). In addition to mindfulness training (e.g., Singh et al., 2011), other related packages targeting skills related to stress reduction and relaxation include behavioral relaxation training (Lundervold et al., 2013). This teaches overt relaxed behaviors through behavioral skill training (e.g., Poppen, 1998) and has been combined with systematic stimulus avoidance assessment to address specific antecedents evoking problem behavior (Wilson et al., 2015).

Another promising approach to relieving stress invokes principles originally discussed in the context of learned helplessness, in which organisms were assumed to learn a passive response to inescapable aversive stimulation (Maier, 1984). Early work in this area suggested organisms learned their escape attempts didn’t matter and therefore they adopted a passive strategy to such aversive situations. In 2016, Maier and Seligman published a retrospective of their learned helplessness theory 50 years after its original publication, updated with new related research. As described earlier, stressful experiences cause a cascade of changes in the body resulting in medical harm (with behavioral situations involving inescapable aversive stimuli doing the most damage). Maier and Seligman (2016) described elements of the neural context that were present during such inescapable aversive stimulation: serotonergic neuronal activity in the dorsal raphe nucleus mediates the learner’s passive response to the inescapable aversive behavioral situation. In this neural context and behavioral situation, escape cannot, and does not, occur (e.g., the pathway inhibits escape). As Maier and Seligman summarize, the passive response turned out to be the default behavioral result of experiencing inescapable stress, instead of being a learned phenomenon. This difference (from the original assumption about passivity being learned, to simply occurring by default during the situation) is crucial: if one can exert behavioral control over the stressor, the stressor ceases to be experienced as an uncontrollable stressful experience, changing the behavioral situation and the underlying neural activity. Given these changes, escape can now occur (and the long-term harm accompanying the stress of passively experienced aversive stimulation can, theoretically, be prevented or reduced).

In 2019, additional research revealed a crucial nuance about the dorsal raphe pathway: the dorsal raphe nucleus serotonergic activity actually switches in function depending on the degree of stress involved in the behavioral situation (Seo et al., 2019). The same neurons switch behavioral functions—from suppressing movement to facilitating movement away from the stressor—depending on whether the stressful events involved are intense and highly threatening to the organism’s safety, or constitute merely a low to medium threat. When the organism can control the threatening stimulus, it becomes less threatening, and escape responses (e.g., movement) can occur (Maier & Seligman, 2016). These new research findings and implications are meaningful to a buffering approach because although aversive stimulation is simply part of the behavioral environment for individuals, one thing therapists and caregivers can program is some degree of control over the aversive stimulus. That is, behavior analysts can assess a client’s behavioral environment for the stressors with which they typically (and are scheduled to) interact, then program experiences in which clients engage in behaviors that detect (e.g., differentially respond to) and control the stressor. This is a reasonable therapeutic intervention both conceptually systematic and theoretically consistent with Maier and Seligman’s (2016) body of work and predicted by subsequent clarifying research on the nature of the dorsal raphe nucleus pathway (Seo et al., 2019). As such, controlling the aversive stimulus and therefore reducing the harmful stress response accompanying it, could prove to be closer than any of the previously described areas to a meaningful, accessible behavioral buffer mitigating harms related to toxic stress. Behavioral control over an aversive stimulus could potentially be functionally related to, and individualized based on, the client’s other behavioral needs, and suggest actions someone can take that (in theory and consistent with Meier & Seligman, 2016) prevent some of the damage related to stress-induced changes in the body. In fact, functional behavioral assessments of behavioral difficulties (such as running away) in children with histories of significant aversive experiences including foster care found that for some individuals, the behaviors were related to gaining access to autonomy and greater control (Clark et al., 2008). This is consistent with reducing severe client behavior indirectly by using a functional contextual approach to enhance enhances client skills involved in differentially responding to and controlling aspects of a previously uncontrollable and unpredictable aversive experience (such as court-ordered interaction with an abusive caregiver).

## Addressing Barriers to Buffer Engagement

Addressing barriers that interfere with accessing or benefiting from buffers may improve health outcomes (and prevent worsening health, especially for those with high rates of adverse childhood and/or conditioning experiences); could improve interactions with clients and their families; may require but also facilitate collaboration with other professionals (see discussion of policy recommendations, below); and may be the right thing to do ethically and behaviorally, given one’s values and the needs of their service population. Some barriers may be behavior and already addressed by the client’s service plan. However, other barriers (such as a lack of information on the importance of buffers, or a lack of connection to providers or resources needed to make the buffers available and accessible) may be simply an artifact of a lack of concerted efforts to install buffers for every client.

Barriers may refer to the risks, response cost, reasons, or resources that result in decreased access to buffers (or to learning about them, engaging in them, or benefiting from them). Learning more about barriers can lead to actions that resolve them: some barriers can be removed, whereas resources must be added in order to solve others. A major benefit of assessing barriers with intention, and as early in services as possible, is the potential for reducing the time a person goes without needed supports. Of course, the buffers may also eventually play more direct roles in reducing difficulties with behavior. Many of the buffers relate to primary sources of reinforcement for clients or caregivers or reflect basic needs that should be met before addressing challenges in other areas (and as such, they may already be covered by assessments the analyst has used to understand the client’s functioning, needs, and health. If not, the behavior analyst might find it helpful to see the attached BTRFLS tool [pronounced “Butterflies”], because it can help to gain client and/or staff or a loved one’s input on issues and needs in each buffer area. (See related policy recommendation where this tool is further described.) For individuals already seeking services from a behavior analyst, the referral process might have highlighted behavioral reasons for missing buffers, such as the lack of important skills necessary to maintain a significant relationship, to regularly prepare and eat nutritious food, or to utilize healthy sleep habits. Because the needs for skill acquisition are often assessed secondary to assessing the needs for behaviors to be reduced or changed, however, many clients of behavioral services will participate in services working on other more pressing needs before the buffer gap is revealed. Prioritizing the buffer areas and working on them earlier in services may have a protective effect.

In addition to the lack of skills supporting engagement in buffers, behavioral barriers include the other kinds of problems regularly solved in ways conceptually consistent with behavior analysis, such as a lack of fluency with respect to the behavior; inadequate stimulus control over behaviors; models for competing behaviors or a lack of models for the more helpful behavior; inadequate or competing contingencies related to the behavior for the person *or for the caregivers who mediate access;* and so on. Alternative or competing behaviors may be more fluent in the repertoire, or be more reinforcing, more available, likely to be prompted, or preferred by others in the home or residential setting. The buffer area may have been “poisoned” or conditioned as aversive (due to previous unfortunate coincidental aversive experience or inappropriate behavioral programming; see Pryor, 2009), or the buffer may be understood or valued in the person’s culture in ways difficult for the analyst to understand or appreciate. It is important that cultural preferences related to each buffer area be explored and approached carefully and compassionately with the individual and family, as no single culture should “own” the buffers or insist on exactly how the buffers should be accessed.

A final behavioral barrier to accessing buffers relates only *indirectly* to the client: there may be a skill acquisition need, but one that occurs in a caregiver or in staff person (e.g., in areas not regularly assessed unless these repertoire gaps directly affect another behavioral problem for the person in services). Apart from behavioral barriers, families and clients also face barriers related to information, resources, or social support, as well as family circumstances-related difficulties requiring assistance in order to solve.

## Policy Recommendations

As suggested above, families and clients may face barriers to accessing support for many of the buffer areas. However, agencies with responsibilities to distribute resources, change behaviors, connect providers, and assess needs may face similar barriers that affect (for example) which clients or caregivers will be offered support, or which needs will be prioritized. One way to reduce the variability in the support agencies offer to clients, caregivers, and their own staff stakeholders is to adopt simple policy related suggestions. Consider this statement: “If there is a group of actions conceptually systematic with respect to behavior analysis, that reduces some of the harmful effects of adverse experiences on the future health of my clients, family, and staff, I am responsible to learn about it and do it.” To what extent is this statement consistent with values the reader (or an organization for which the reader works) already holds dear? An agency that answers this question in the affirmative might next conduct an options analysis for adding a buffer policy, then adopt related policy if the potential benefits significantly outweigh potential risks.

### Establish a Collaborative Provider and Resource Network

Providers interested in beginning to support more of their clients in a concerted way across all six buffer areas may need to begin, join, or grow a collaborative provider network whose members are available to problem solve, provide information, make and complete referrals, and address barriers. This may have benefits to agencies, individuals, and families, but it also addresses one of the most meaningful reasons agencies ignore buffer work: they don’t know how to do it themselves, or lack a group of providers and agencies with whom they can partner when needed. Establishing a collaborative network is especially useful in the event the behavioral agency lacks in-house some of the resources clients and caregivers would need to facilitate growth in all six buffering areas. Such a network makes it more likely that each behavioral provider has access to connections for any specialty necessary for referrals for information, support, or resources to identify or problem solve ways to reduce barriers to engagement in buffering areas.

### Adopt a Preventive Information Policy on Buffers and Barriers

It is recommended to adopt a policy that makes information on buffers and solving barriers available as an antecedent to client services. Just as it is helpful to educate clients and families on the analyst’s scope and competence, company policies, and ethical guidelines before beginning services, care should be taken to provide buffers related information before it becomes an issue. This standardizes and makes available the answers (for both staff and families) to such questions as, what are the conditions under which clients, caregivers and staff learn about buffers? Is this information provided only as a consequence to a problem, or does preventative education take place before problems occur as part of setting up conditions for a client’s success? Does information come from sources behavior analysts are likely to distrust or ignore because of perceived incompatibility with their work? In terms of resource barriers, do those in need of buffers have access to funding streams that could cover education and the cost of problem solving in order to make buffer engagement possible? Is transportation a barrier for the family to get to services, and does the family have the time needed to pursue them?

**Fig. 1:**
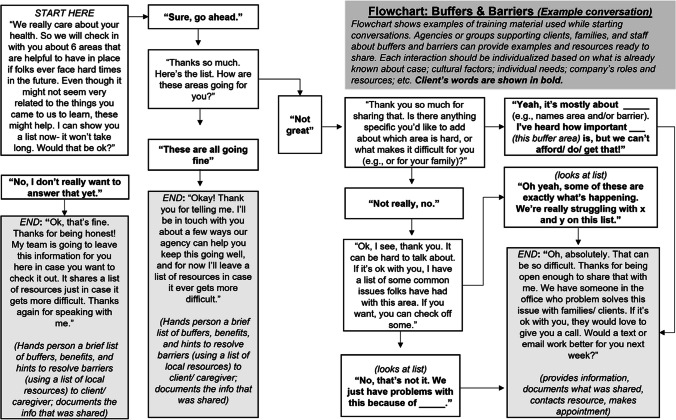
Example conversation to reduce response effort for practitioners new to adopting buffers policy (author original graphic)

**Fig. 2:**
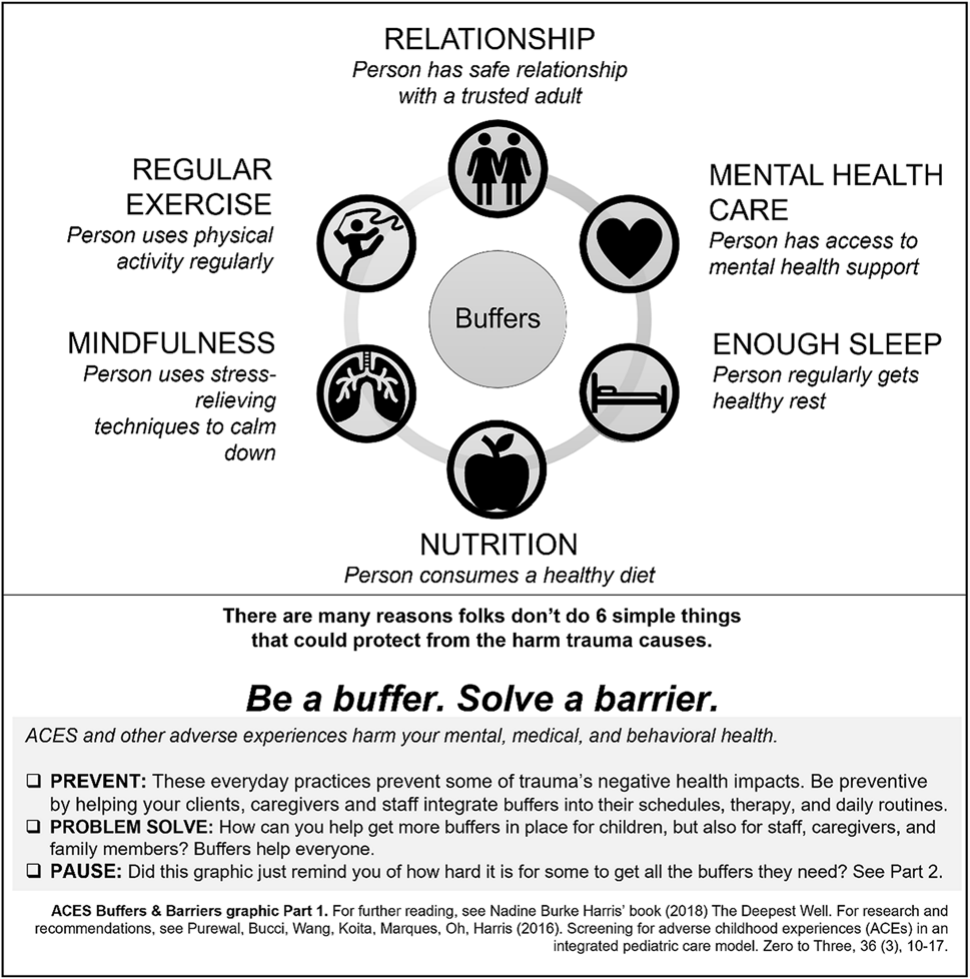
ACES buffers and barriers graphic part 1: Buffers (author original graphic)

**Fig. 3:**
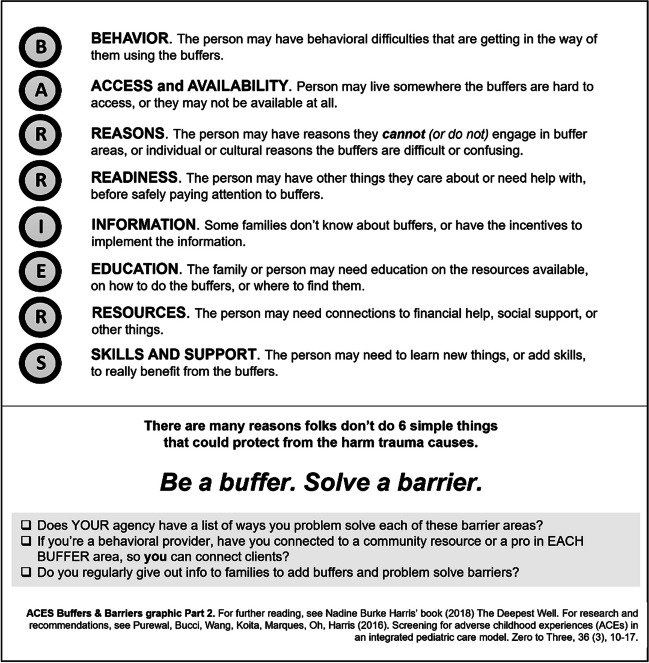
ACES buffers and barriers graphic part 2: Barriers (author original graphic)

Related to this recommendation, samples of basic informational handouts are provided in this article, beginning with a simple conversation with a family or client (see Fig. 1, **“**Example Conversation to Reduce Response Effort for Practitioners New to Adopting Buffers Policy”) to learn more about a new client’s access to and engagement in buffering areas. When the idea of buffers is completely novel to staff and clients, introducing the idea using a visual like those found in Figs. 2 and 3 may be of assistance, and it is encouraged to allow these examples to inspire making or accessing additional graphics more specific and appropriate to individual clients, communities, or cultures. Later in supports, it might be productive to dive more deeply into understanding barriers to benefiting fully from engagement in a particular buffer area.

### Assess Repertoires and Environments for Presence of Skills and Supports Related to Buffer Areas

**Fig. 4:**
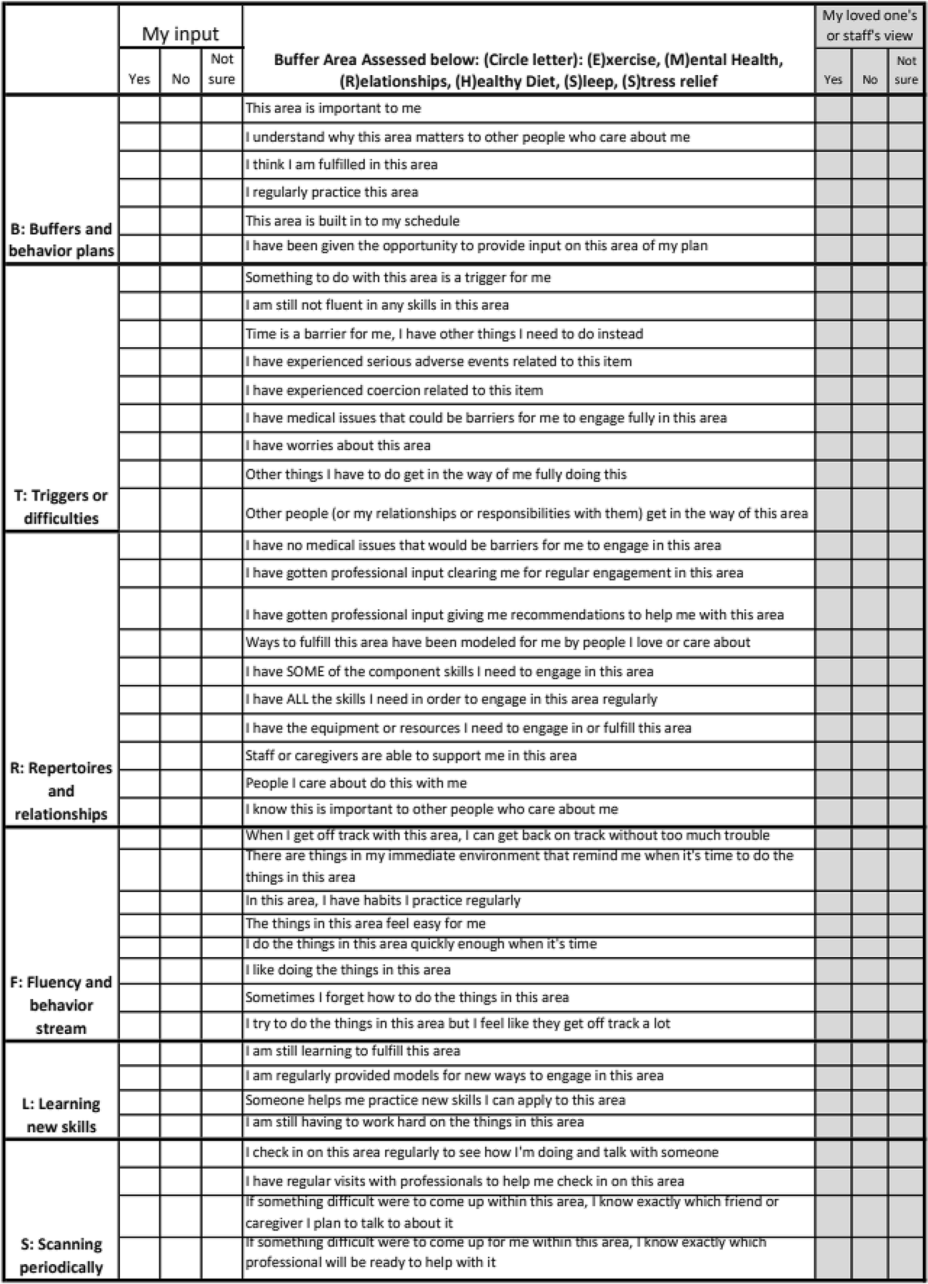
BTRFLS (pronounced “butterflies”): This visual provides a quick glance at some barriers clients may want to discuss related to their engagement in buffering areas

Each of the buffer areas relates to accessing reinforcers in the client’s life. Thus, clients should have an array of appetitive, safe, accessible, and engaging alternative behaviors that meet the needs related to each buffer area. One way to ensure this is the case is to periodically scan environments for elements that contribute to or hinder their engagement or access to the buffer areas, and to regularly document how the team is working on improving access or problem-solving other issues together with the client. To this end, the BTRFLS (pronounced “Butterflies”) tool is described below (see Fig. 4), in the context of suggestions for examining buffers relevant to clients. As always, individuals should be provided as much autonomy as possible, and be fully involved from the beginning (e.g., examining whether the buffers are present and what it might mean to the client) to maintenance (e.g., practicing the buffers). Questions and issues may not all be relevant for a given individual, and delivery should be adapted depending on the client’s skills, history and support. Some of this information may be available by observing the client and their routines or reviewing interviews with the client, caregiver or familiar therapists.

#### Using the BTRFLS Tool to Examine Buffers and Barriers with Clients and Families

“B” is for buffers and behavior plans. First, note whether the person currently engages in the buffer area. A client or team member notes whether the area is already important to the person (e.g., connected to their values in a way they understand and endorse) and whether it matters to others in their life. If the person does not yet regularly engage in the area but is working on it with their behavioral providers, the team might prioritize questions like “Does the person regularly engage in practicing the area, and is it built into their schedule? Does the person have regular opportunities to provide input on this area of their behavior plan or life?”

“T” stands for triggers or difficulties. At this point, the provider determines whether the buffer area is related to current difficulties. For instance, is something related to this buffer area considered a “trigger” (e.g., related to subsequent difficult responses the client often experiences when attempting to engage in the buffer area)? Other difficulties might be a lack of fluency in related skills, a lack of time to engage in the area, a history of experiencing aversive events or coercion related to the area, the presence of medical issues that might be barriers to engaging in the area, worries the client expresses about the area, or people who prevent the client from engaging in the area.

“R” stands for repertoires and relationships. After assessing the client’s experiences of difficulties related to the area, the behavior analyst might consider how the client’s relationships and existing repertoires can be leveraged to support the client’s engagement in the area. Has the client received professional support, staff support, or caregiver help in this area? Do individuals the client cares about model how to engage in this area and/or help by joining the client to do these activities? Does the client have the skills (including component skills) and the resources and equipment they need to engage in the area?

“F” stands for fluency and the behavior stream. At this point, the behavior analyst considers whether the client has skills necessary to switch back to healthy engaging in the area. Does the client seem to enjoy doing these things and do them with ease, and do they have habits that support regular engagement? Do distractions side-track the client from doing these things (and when needed, does the behavioral environment already hold the discriminative stimuli, motivating operations and prompts that assist the client to jump back in to practicing this buffer)?

“L” stands for learning new skills. In some areas, the client may still be in a phase of skill acquisition. If this is the case, are there regular models provided by people the client cares about, and someone to help the client regularly practice?

“S” stands for scanning periodically. Even if the client has practiced most buffer areas in the past, it is still helpful to check in regularly to make sure things have not shifted in a problematic way. For some areas, regular visits with professionals may be needed (such as a check-in with a mental health provider that the client can look forward to even if nothing is “wrong,” or a periodic session with a nutritionist to be exposed to new foods and recipes in the buffer area of nutrition, or a session with a personal trainer to model activity sampling appropriate to the client’s fitness level and physical needs). If there were to be a problem in a buffer area, does the client have someone identified in their personal life who is always ready for the client to contact them about this; does the client (or caregiver) have the contact information for the team provider best suited to provide support in a professional capacity related to a given buffer area?

Overall, if agencies and clients and their families agree that buffers are a priority, they will be better equipped to problem solve together, because some of the barriers (like having the time needed to pursue buffer engagement) can be addressed by interaction with partners. For instance, when an agency policy provides for the integration of buffer areas in each client’s support plan, a family experiences less of a burden to work on that buffer in the home setting, whereas the potential for experiencing benefits related to that particular area may be enhanced.

## General Recommendations

General recommendations for interested practitioners are to utilize technology and to begin strategically. In the current climate, one recommendation for the research community is to emphasize the exploration of technology options already available for phone or tablet application, online, or text-based programs improving participation in the buffer areas, using a comprehensive approach addressing multiple buffers. By itself or in combination with a few others, many of the buffer areas have already been targeted in internet-based delivery of behavioral programming to support engagement at the individual or community level, with increasing acceptability of such methods (e.g., see Winett et al., 2007 on nutrition and physical activity; see McLay et al., 2020, for telehealth delivered treatment of sleep problems; for mental health, see Charbonnier et al., 2022; for online delivery of programs providing acceptance and commitment therapy and parent training, see Andrews et al., 2021). There exist many downloadable and/or wearable user applications assisting with tracking and monitoring personal data, as well as managing and receiving content or recommendations related to nutrition (e.g., Fooducate), fitness (e.g., Caliber), mental health and therapy (e.g., Talkspace), sleep (e.g., Sleep Cycle), and mindfulness and meditation (e.g., Headspace), often in some combination and costing between a few to hundreds of dollars per year (Sayer, 2023).

Thus, it is suggested that attention be paid to integrating buffer areas in a preventive and comprehensive format to target individuals, families or community groups using the means they are already familiar with and likely to access.

In asking where to start, one needs to know which persons are most at risk in the absence of buffers. Lanier et al. (2018) found that a high number of ACES increased children’s risk for poor health outcomes, an anticipated outcome of their research and consistent with other studies. It is critical that this study also revealed that one combination in particular—experiencing both poverty and a caregiver with mental illness—was significantly more dangerous in terms of poor health outcomes for children, even if those were the only two adverse experiences reported. Although research on specific combinations of adverse experiences is in a fledgling state, this finding suggests that clinicians interested in prioritizing and directing their limited resources might do well to aim prevention efforts at the most vulnerable groups of clients and caregivers in their care (especially if they cannot yet justify installing every buffer, for every client family).

Which buffer areas would be most helpful to target first? For researchers or clinicians interested in prevention, one locus for intervention may be the interconnections between buffer areas, and among the people involved in facilitating them. Many (if not all) of the buffer areas described here are interrelated: consider, for example, the connections between having a healthy relationship, and getting adequate sleep, nutrition, exercise, and mental health care; or the research-based connection between exercise, healthy eating, and getting enough sleep. In a study with adults with mental illness and poor health, Schmutte et al. (2018) found that after a 14-week self-management program targeting sleep, participants not only experienced significantly improved sleep quality, but also reported increases in healthy diet and exercise.

In addition to sleep, the buffer area involving mindfulness and stress reduction may be a particularly beneficial one to target initially. There is a strong relationship between *behavior*, the purview of behavior analysis, and parent psychological distress, much less studied by behavior analysts although it is an area valuable to questions of social validity and acceptance of treatment. Research shows that even more than core aspects of client impairment (such as the client’s cognitive abilities), behavior problems play huge roles in a parent’s psychological distress (Lecavalier, 2006; Lounds et al., 2007). However, programs that integrate mindfulness with positive behavior support training for parents have been shown to improve parent management of challenging behaviors while producing significant reductions in the parents’ stress (Singh et al., 2014).

Finally, in considering buffers as a preventive measure that reduces exposure to harms related to adverse experiences, it may be crucial to suggest buffers for *stakeholders of behavioral services* rather than for the more limited group often referred to as “clients.” Consider a hypothetical policy in which several types of stakeholders (for instance, a group home agency’s staff, the behavior analysts, caregivers and parents, and clients) are *all* taught to use mindfulness, and are supported to engage regularly in healthy diets, a mindfulness practice, mental health support, an exercise program, healthy sleep habits (and to value and pursue safe warm relationships). How would one quantify the benefits of such a program, and would the benefits be worth the time spent developing it?

## Conclusion

Delivering behavior analysis in such a way that improves public health has significant precedent in the literature (Alligood & Gravina, 2020; Biglan et al., 2000; Biglan et al., 2020; Hovell et al., 1986; Fisher et al., 2011; LeBlanc et al., 2020; Normand et al., 2021; Winett et al., 1991). The six buffer areas discussed herein reflect several worldwide health priorities, and converge on a possible preventive locus of behavioral action that could mitigate harm resulting from adverse experiences. As behavior analysts utilize their own repertoires to enhance the efforts of others, they can solve issues meaningful to a community organization or family. Consultation can be provided for an individual, but also delivered via telehealth and/or in brief informational sessions with agencies interested in (but unsure how to best incorporate) committed actions in buffering areas. Community-oriented preventive supports are critical to pair with information covering how and when to locate services when more individualized behavioral consultation is warranted. This could provide community members with knowledge on the scope of practice for a behavior analyst, resources on connecting with other professionals, and something else invaluable: positive and useful experiences with behavioral providers, not predicated on someone’s need to reduce behavior.

## Data Availability

Data sharing is not applicable as no datasets were generated or analyzed for the purposes of this article.

## Declarations

### Protective Factors and Buffering:

Values driven policy that can address adverse childhood experiences and trauma
